# Is a tiered restrictions system an effective intervention for COVID-19 control? Results from Portugal, November-December 2020

**DOI:** 10.1186/s12889-024-18369-1

**Published:** 2024-04-04

**Authors:** Marta Moniz, Patrícia Soares, Baltazar Nunes, Andreia Leite

**Affiliations:** 1 Public Health Research Center, Comprehensive Health Research Center, NOVA National School of Public Health, NOVA University Lisbon, CHRC, Lisbon, Portugal; 2 Centre for Vectors and Infectious Diseases Research, National Institute of Health Doutor Ricardo Jorge, Águas de Moura, Portugal; 3 Department of Epidemiology, National Institute of Health Doutor Ricardo Jorge, Lisbon, Portugal

**Keywords:** COVID-19, Tiered restrictions, Incidence rate, Infectious diseases, Epidemiology

## Abstract

**Background:**

In November 2020, similar to other European countries, Portugal implemented a tiered restrictions system to control the COVID-19 pandemic. We aimed to compare the COVID-19 growth rate across tiers to assess the effect of a tiered restrictions system in Portugal, using models with different times between tiers assessment. Our hypothesis was that being in a higher tier brings a faster deceleration in the growth rate than being in a lower tier.

**Methods:**

The national database of notified COVID-19 cases and publicly available data were used to analyse the effect of the tiered restrictions system on the COVID-19 incidence growth rate. The tiers were based on the European Centre for Disease Control risk classification: moderate, high, very and extremely high. We used a generalised mixed-effects regression model to estimate the growth rate ratio (GRR) for each tier, comparing the growth rates of higher tiers using moderate tier as reference. Three models were fitted using different times between tiers assessment, separated by 14 days.

**Results:**

We included 156 034 cases. Very high tier was the most frequent combination in all the three moments assessed (21.2%), and almost 50% of the municipalities never changed tier during the study period. Immediately after the tiers implementation, a reduction was identified in the municipalities in high tier (GRR high tier: 0.90 [95%CI: 0.79; 1.02]) and very high tier (GRR very high tier: 0.68 [95%CI: 0.61; 0.77]), however with some imprecision in the 95% confidence interval for the high tier. A reduction in very high tier growth rate was identified two weeks (GRR: 0.79 [95%CI: 0.71; 0.88]) and four weeks (GRR: 0.77 [95%CI: 0.74; 0.82]) after the implementation, compared to moderate tier. In high tier, a reduction was also identified in both times, although smaller.

**Conclusions:**

We observed a reduction in the growth rate in very high tier after the tiered restriction system was implemented, but we also observed a lag between tiered restriction system implementation and the onset of consequent effects. This could suggest the importance of early implementation of stricter measures for pandemic control. Thus, studies analysing a broader period of time are needed.

**Supplementary Information:**

The online version contains supplementary material available at 10.1186/s12889-024-18369-1.

## Background

Portugal experienced the first wave of COVID-19 cases from March to June 2020 [1], with a national lockdown in place from 18th March to 3rd May [2], requiring citizens to stay at home except to access medical care, daily exercise, shopping for essentials, and essential work travel. September 2020 saw a resurgence of COVID-19 cases, with a rapid increase in the number of new cases, which coincided with the return to face-to-face work and school after the summer holidays [1]. This second wave was unevenly distributed across the country, with certain areas more severely affected than others, particularly in the metropolitan areas and their adjacent municipalities [1].

Lockdowns started to be implemented worldwide to mainly contain deaths and prevent healthcare systems from becoming overwhelmed and thus hampered from helping those in need [3]. However, this approach had profound consequences on people’s lives [4, 5], namely negative social and economic impacts [4–6]. A study compared the health-related quality of life (HRQoL) of Portuguese citizens during the COVID-19 pandemic to pre-COVID-19 (data between November 2015 and January 2016) and found that HRQoL decreased during the pandemic [4]. Furthermore, not only HRQoL but also cancellation or postponement of medical appointments differed between sex, age groups and economic levels [4] Rearrangements related to household configuration also happened as a way to cope with restrictions, namely young adults moving to their parents’ house [5]. In Portugal, it was estimated that, during the first lockdown, the poverty rate increased by 4%, and without the government mitigating policies, it would have increased by 20% [6]. Additionally, it was estimated that people lost 7% of their annual income during the first lockdown [6]. These losses were also different geographically and between professional groups [6]. However, although decreasing transmission remained paramount to control the pandemic, lockdown social consequences were dooming societies all over the world. Hence, tiered measures started attaining support as an alternative to national lockdowns, adapting public health measures to the risk needs of each population geographically.

Following other European countries [7, 8], Portugal adopted tiered measures to control the COVID-19 pandemic on 9th November 2020, targeting the municipalities that were at higher risk, considering the European Centre for Disease Control (ECDC) guidelines (> 240 cases/100,000 inhabitants) [9]. Later, on 24th November, the country adopted a four-tiered restriction system nationwide [10]. Each tier was defined according to the European Centre for Disease Control (ECDC) risk categorisation: moderate (< 240 cases/100 000 inhabitants); high (240–480 cases/100 000 inhabitants); very high (480–960 cases/100 000 inhabitants); and extremely high (> 960 cases/100 000 inhabitants) [11]Tiers were assigned on a municipality level, only according to COVID-19 14-day cumulative incidence. The situation was monitored every two weeks using 14-day cumulative incidence estimates at the municipality level at the beginning of the week. Certain measures were applied to the entire country, but others depended on the municipality’s tier. Measures applied nationwide were incorporated in the moderate tier. On each two-week assessment, municipalities could move down to a lower tier (if not moderate), move up to a higher tier (if not very or extremely high), or maintain the tier previously assigned. As the tier increased, the measures were cumulatively added. Measures of moderate tier, enforced nationwide, were mainly mandatory use of masks, prevention of social gatherings by closing establishments (i.e. bars and restaurants) and prohibition of circulation outside municipalities on the eve of public holidays. High tier added mandatory curfews, mandatory teleworking and earlier closure of establishments. Very and extremely high tiers added earlier and more days of mandatory curfew, general duty of confinement outside the period of mandatory curfew, closure of commercial establishments and suspension of retail activities on the eve of public holidays. A non-exhaustive list of the main measures applied in each tier is presented in Additional File 1, and the detailed measures can be found in the Portuguese official sources [9, 12]. After 22nd December, although the tiered system was still in place, the measures were relaxed due to the holiday season [13], and on 15th January 2021, a national lockdown was enforced [14].

The evidence available, from other European countries, on the impact of similar tiered restrictions systems on COVID-19 transmissibility suggests a decrease in the effective reproduction number R(t) in all tiers, with a larger decrease of COVID-19 cases in tiers with stricter measures [7, 8, 15–18], compared to those with less strict ones. The Italian tiered system was a progressive colour-coded system implemented on a regional basis, where tiers were based on the combination of 21 quantitative indicators on the level of transmission, the burden on older age groups and healthcare, and the resilience of monitoring systems [18, 19]. This study showed that this tiered system decreased human activities [7], namely time spent outside home, public transportation, recreational and retail activities and also decreased contacts [18], with stricter tiers showing a higher decrease. However, the decrease in the time spent outside home observed in all tiers was below the values observed during the lockdown [7]. Transmission also decreased, with R(t) values below 1 in all tiers, but the decrease was sharper in stricter tiers, with stricter measures related to people’s movement and gatherings [7, 18]. The United Kingdom tier system was a progressive three tier system based on transmission levels, infection growth rates, age distributions, and the capacity of local healthcare services [20]. Several studies analysed the impact of the tier system in this country, but the evidence appears to be mixed. Although tier 1 and 2 showed a minor reduction in transmission or contact rates, tier 3 (stricter) seems to have reduced both rates [8, 15–17] with no differential effects by socioeconomic groups [15].

Despite the evidence from these European countries, the literature available regarding the tiered restriction system is still limited. Of the European countries we could find evidence from the tiered system intervention, Portugal was the only one that used the ECDC risk criteria alone for tiers assignment and analysed COVID-19 incidence growth rate variation in each tier. This study adds to the existing literature on tiered measures, providing evidence of these criteria.

To our knowledge, only one study analysed the implementation of tiered measures in Portugal. In that study, COVID-19 cases pre- and post-restrictive non-pharmaceutical interventions (NPIs), implemented at different times in different regions, were evaluated to analyse the impact of tiered NPIs in reducing COVID-19 incidence. The results suggest that both stringency and time under the stricter measures contributed to revert the increasing COVID-19 incidence trend [21]. Although the study found a decline in COVID-19 cases with the implementation of tiered measures, it did not explore differences between tiers. Nonetheless, it was the first study describing the effect of implementing tiered measures in Portugal. The goal herein was to analyse the impact of the nationwide tiered system on COVID-19 growth rate, based on municipality risk level, thus adding evidence regarding this kind of tiered interventions using the experience from Portugal. This study sheds light on the effect of tiered measures on the growth rate of COVID-19, which was not examined before. Implementing tier measures could be a valuable tool in controlling respiratory pandemics, potentially preventing the socioeconomic repercussions of nationwide lockdowns. Here, we compared the COVID-19 growth rate across tiers to assess the effect of a tiered restrictions system in Portugal, using models with different times between tiers assessment. Similar to what was observed in the abovementioned studies, where stricter tiers had a higher decrease in COVID-19 infection, we hypothesised that being in a higher tier brings a faster deceleration in the growth rate than in a lower tier.

## Methods

### Study period and study design

We conducted an ecological study to analyse the effect of the tiered restrictions system on COVID-19 incidence growth rate in Portugal, using the national database of notified cases.

The tiered restrictions system was implemented on 24th November 2020 (Tuesday), based on the 14-day cumulative incidence reported on the 23^rd of^ November report, released on Monday. Since the reports were only available on Mondays and the measures implemented were based on that information, our study period ranged from 23rd November and 21st December 2020. We did not consider a longer period since, on 22nd December, measures were relaxed, and then replaced by a national lockdown in January 2021. The lockdown was continuously renewed until April 2021, when a 4-phase deconfinement plan began to be implemented [22]. Within the study period, there were three tiers’ assessments, separated by 14 days: 23rd November (T1), 7th December (T2) and 21st December (T3). T1 corresponds to the beginning of the tiered restrictions system implementation and the first tier assessment, T2 corresponds to the second tier assessment and T3 to the ending and the third tier assessment. Although tiers were assessed based on the 14-day cumulative incidence, we calculated a 7-day cumulative incidence to observe weekly changes since these could take longer to observe considering a 14-day cumulative incidence.

**Fig. 1:**
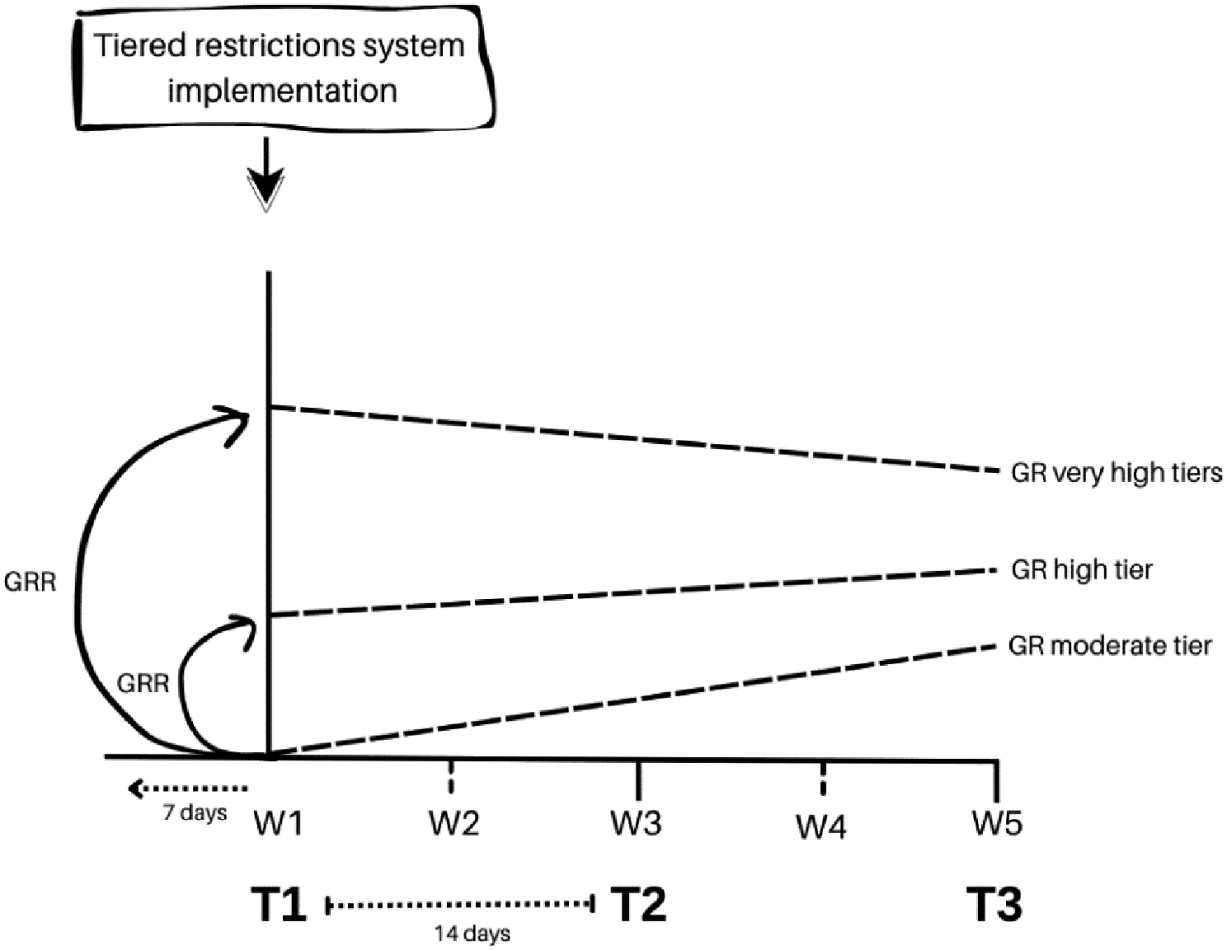
Study design. T1– Beginning of tiered restrictions system; T2– Change tier; T3– Change tier and ending of tiered restrictions system; GR– Growth rate calculated between each time point (W1-W5); GRR– Growth rate ratio calculated between T1-T2, T2-T3 and T1-T3; 7-day cumulative incidence calculated each week considering 7 days before

Figure 1 depicts our overall design. The study period was divided in intervals of 7 days, which resulted in five time points: W1 (23-11-2020), W2 (30-11-2020), W3 (07-12-2020), W4 (14-12-2020), W5 (21-12-2020). At each time point (Mondays), we assessed the corresponding 7-day cumulative incidence starting the prior week, i.e., W1 incidence was given by the 7-day cumulative incidence between 17th November and 23rd November. The incidence in these time points was then considered to calculate the growth rates between each point in time, referred to as GR in Fig. 1. The incidence rate (IR) refers to the rate of COVID-19 cases in moderate tiers at each time point. We also calculated the incidence rate ratio (IRR), i.e., the incidence rate variation in higher tiers compared to the moderate tier. The growth rate (GR) estimated the rate variation implemented by the tiered system, and GRs were compared using a growth rate ratio– our effect measure, referred to as GRR in Fig. 1. This measure estimated the effect of implementing a higher versus a lower tier, that is, the variation in growth rate observed in the higher tiers after the tier system implementation, compared to the growth rate in the moderate tier (reference tier). A comprehensive explanation of the analysis rationale can be found in the statistical analysis section.

Since the measures enforced in very high and extremely high tiers were the same, we merged these two tiers into one tier, “very/extremely high”, which will be referred to as “very high tier”.

### Data sources and variables

Three data sources were used. We used the reports from the Portugal Directorate-General of Health (DGS) website [23] to retrieve the tiers assigned to each municipality when tiers were assessed (23-22-2020; 07-12-2020; 21-12-2020). We extracted the daily number of cases per municipality from the national COVID-19 database [24], considering the notification date as the case date, which had information on the number of all the daily confirmed COVID-19 cases by RT-PCR test since the first COVID-19 case notification, per notification date. Population estimates for each municipality in 2020 were retrieved from the Statistics Portugal site [25], including data from 308 municipalities (278 in mainland, 11 in Madeira and 19 in Azores). Cases with no assigned municipality were excluded.

### Statistical analysis

We first provided a description of the tier’s implementation. This included mapping the tiers assessed at each time point and describing the possible combinations of tiers in the three points assessed alongside their relative frequency. The latter was intended to support the interpretation of our measures, given that municipalities could be assigned the same tier on the three data points or different tiers depending on the epidemiological evaluation. We also provided a visualisation of the 7-day cumulative incidence mean by tier.

To estimate the GRR for each tier assessment a generalised linear mixed-effects model with a negative binomial distribution was used as follows:

$$ \begin{aligned}\text{l}\text{o}\text{g}\left(E\left({Y}_{m,w}\right)\right)&= {\beta }_{0}+ {\beta }_{1}* week+ {\beta }_{2}* {Tier high}_{m,w}\\&\quad+ {\beta }_{3}* {Tier very high}_{m,w}+ {\beta }_{4}* {Tier high}_{m,w}*week\\&\quad +{\beta }_{5}* {Tier very high}_{m,w}*week+\text{l}\text{o}\text{g}\left(p\right)+{\epsilon }_{m,w}+ {u}_{m} \end{aligned}$$

Where: *week* corresponded to each time point (W1-W5); *tier* corresponded to the tier (moderate, high, very high) that each municipality was assigned in each time point; $$ {u}_{m}$$ corresponded to the random effect for municipality *m*; $$ {Y}_{m,w} $$is the 7-day cumulative number of COVID-19 cases in municipality *m* in week *w; exp(*$$ {\beta }_{1})$$ corresponded to the weekly growth rate in municipalities classified as moderate tier (reference); $$ {\text{e}\text{x}\text{p}(\beta }_{2})$$ and exp($$ {\beta }_{3})$$ corresponded to the incidence rate ratio of municipalities in high and very high tiers, respectively, versus municipalities in moderate tier; and exp($$ {\beta }_{4})$$ and exp($$ {\beta }_{5})$$ corresponded to the GRR of municipalities, *m*, in high and very high tiers, respectively, versus moderate tier in each week *w*. Hence, the measures of interest were $$ {exp(\beta }_{4})$$ and $$ {exp(\beta }_{5})$$, which gave our GRR. As a further description, we also provided $$ exp({\beta }_{1}+ {\beta }_{4})$$ and $$ {exp(\beta }_{1}+ {\beta }_{5})$$, which gave us the weekly growth rate at high and very high tiers. The population of each municipality was used as the offset variable.

We performed three models to analyse the effect of the tiered restrictions system on the incidence growth rate at various times:

- Model 1: implementation– focused on the timing of tier implementation and was intended to provide the immediate effect of the tiered restrictions system implementation. We thus assessed the time between the first tier assessment (T1) and the second time tier assessment (T2), by incorporating the first three time points (W1, W2, W3). Therefore, and in accordance with our regression model, the immediate effect of the tiered restrictions system implementation, i.e., the GRR of higher tiers versus moderate tier, was given by ($\text{e}\text{x}\text{p}\left({\beta }_{4}\right)$) for the high tiers and by ($\text{e}\text{x}\text{p}({\beta }_{5}$)) for the very high tier of model 1.
- Model 2: change– was meant to assess the effect of the measures at the second tier assessment (T2) and thus comprised the time from the second tier assessment (T2) to the last tier assessment (T3), by incorporating the remaining three time points (W3, W4, W5). The measures of effect were similar to the abovementioned but provided the effect of tiered measures renewed at T2.
- Model 3: overall– provided an overall effect of the tier system implemented during approximately one month, considering two tiers’ assessments and corresponding measures. It comprehended the time from when the tiers were first implemented (T1) to the last tier assessment (T3), including all the time points (W1, W2, W3, W4, W5). The measures of effect were similar to the abovementioned but provide a distinct effect. We performed two sensitivity analysis associated with the study design: (i) considering a 14-day cumulative incidence to analyse the effect of a longer period on COVID-19 growth rate and observe if the effects of the tiered measures could take longer to emerge; (ii) separating the tiers very and extremely high, performing the analysis considering four tiers (moderate, high, very high, extremely high). This analysis was performed to understand the separate effect of these tiers. Although the measures implemented were the same between very high and extremely high tiers, individuals might have a different risk perception and change their behaviour, consequently affecting COVID-19 growth rate. We also tested our hypothesis that the growth ratio was tier-dependent, eliminating the interaction between tier and time to compare with our initial hypothesis that the growth ratio was tier dependent. Additionally, we considered municipality as a fixed variable to assess the assumption that the municipality also had different growth rates. All statistical analyses were performed using R 4.2.2 [26].

## Results

We included 156 034 COVID-19 cases distributed in 308 municipalities and excluded 832 cases with no assigned municipality for the period analysed. At the beginning of the tiered restrictions system (T1), 95 (31%) municipalities were in moderate tier, 87 (28%) in high tier, and 126 (41%) in tiers very high. In the second assessment (T2), 100 (32%) municipalities were in moderate tier, 94 (31%) in high tier and 114 (37%) in tiers very high. Finally, at the third assessment and the end of the tiered system restrictions (T3), 104 (34%) municipalities were in moderate tier, 95 (31%) in high tier, and 109 (35%) in very high tier.

### Tiers assessment description

**Fig. 2:**
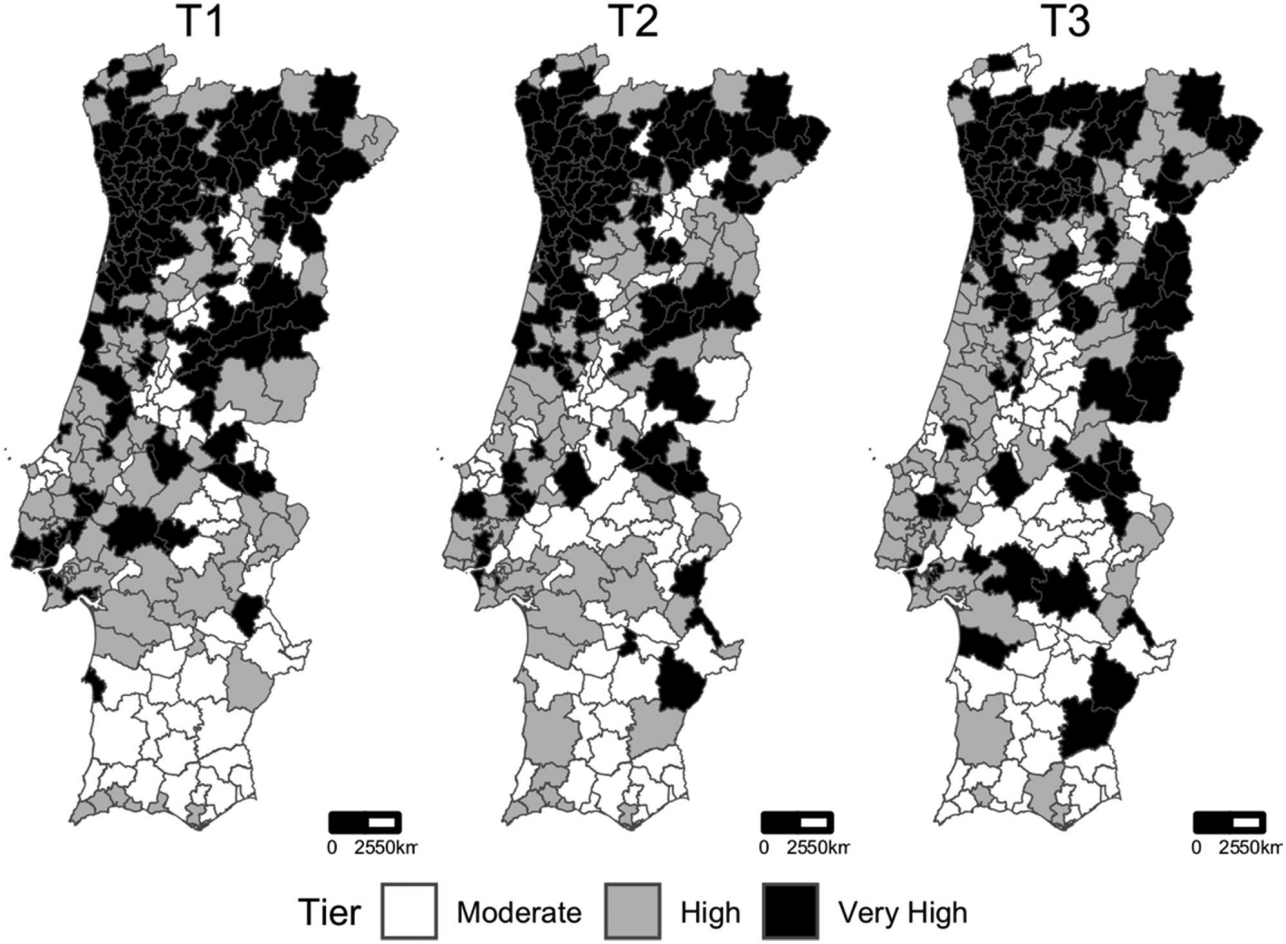
Geographic distribution of tiers in each moment of tier assessment. Azores and Madeira islands are not visually represented due to space issues. T1: 21-11-2020; T2: 07.12.2020; T3: 21.12.2020

As shown by Fig. 2, the North region was initially (at T1) the region that concentrated the municipalities at the highest risk tier. Over time, the infection started to spread to other regions, namely to more inland and southern regions of the country, as shown by the spread of the darkest spots on the maps, corresponding to very high tier. For Azores and Madeira, the majority of municipalities stayed in moderate tier during the entire study period (data not shown).

Tiers in the three moments assessed combined in 26 different patterns (Table 1). The most frequent combination was municipalities in very high tier in all the three moments assessed (21.2%), followed by municipalities that remained in moderate tier in the three tiers assessment (18.6%). Almost 50% of the municipalities never changed tier during the study period.

**Table 1:** Tiers combination in each moment of tier assessment (T1, T2, T3)

Tiers				
T1	T2	T3	N	%
Very High	Very High	Very High	65	21.1
Moderate	Moderate	Moderate	59	19.2
High	High	High	24	7.8
Very High	Very High	High	22	7.1
Very High	High	High	18	5.8
High	High	Very High	14	4.5
High	High	Moderate	12	3.9
Moderate	Moderate	High	11	3.6
High	Very High	Very High	11	3.6
High	Moderate	Moderate	10	3.2
Very High	Moderate	Moderate	7	2.3
Moderate	High	High	6	1.9
High	Moderate	High	6	1.9
Moderate	High	Moderate	5	1.6
Moderate	High	Very High	5	1.6
High	Very High	High	5	1.6
Very High	High	Moderate	5	1.6
Very High	High	Very High	5	1.6
Moderate	Moderate	Very High	3	1
Moderate	Very High	Very High	3	1
High	Moderate	Very High	3	1
Very High	Very High	Moderate	3	1
Moderate	Very High	High	2	0.6
High	Very High	Moderate	2	0.6
Moderate	Very High	Moderate	1	0.3
Very High	Moderate	High	1	0.3

### Incidence and growth rate description

**Fig. 3:**
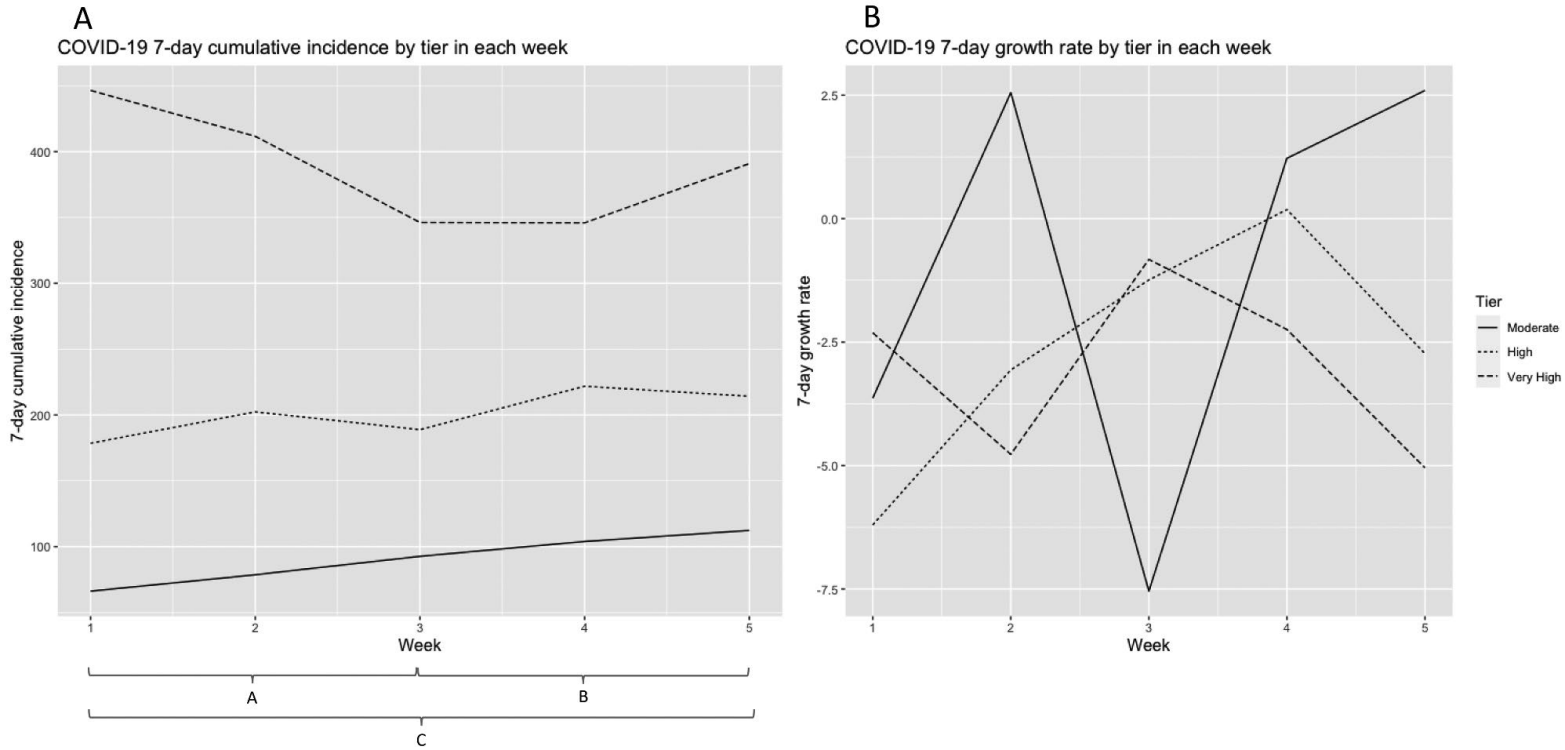
**3 A**. COVID-19 7-day cumulative incidence mean at the beginning of each week (W1-W5) by tier; **3B**. COVID-19 7-day growth rate mean at the beginning of each week (W1-W5) by tier. A– weeks used in model 1; B– weeks used in model 2; C– weeks used in the full model

Figure 3 shows the current epidemiological situation (panel A) and the temporal dimension (panel B). These panels are presented combined to facilitate the understanding of the effect of the tiered measures. During the period analysed, the mean COVID-19 cumulative incidence (Fig. 3A) increased in moderate tiers during the study period. The incidence in high tier peaked in W4, between the first and second tier assessments. In very high tier, the incidence decreased until W3 (second tier assessment) and then increased until W5, the third tier assessment and the end of the tiered restrictions system. The mean growth rate (Fig. 3B) in the moderate tier had a more pronounced peak between the first and second assessment (W3) but remained heterogeneous throughout the study period. In the high tier, the growth rate continued to increase until W4, after the second tier assessment. The growth rate in the very high tier decreased right after the first tier assessment (W1) but after the second assessment (W3), the decrease became more stable.

### Models analysis

**Table 2:** COVID-19 incidence trends after the tiered restriction system implementation, by tier, considering different time periods

Parameters	Model 1 (T1-T2)		Model 2 (T2-T3)		Model 3 (T1-T3)	
	Estimate	95% CI	Estimate	95% CI	Estimate	95% CI
IR in Moderate tier in the first moment*	56.92	[45.12; 71.81]	41.03	[27.93; 60.27]	63.94	[53.76; 76.05]
IRR High tier vs. moderate tier	2.66	[1.96; 3.63]	3.44	[2.03; 5.85]	2.38	[1.91; 2.98]
IRR Very high tier vs. moderate tier	8.29	[6.20; 10.90]	6.98	[4.31; 11.31]	5.82	[4.71; 7.18]
GR Moderate tier	1.18	[1.07; 1.30]	1.23	[1.12; 1.34]	1.15	[1.10; 1.20]
GR in High tier	1.06	[0.98; 1.15]	1.08	[1.00; 1.17]	1.06	[1.02; 1.10]
GR in Very high tier	0.81	[0.76; 0.86]	0.97	[0.91; 1.04]	0.89	[0.87; 0.92]
GRR High tier vs. moderate tier	0.90	[0.79; 1.02]	0.88	[0.78; 1.00]	0.92	[0.87; 0.98]
GRR Very high tier vs. moderate tier	0.68	[0.61; 0.77]	0.79	[0.71; 0.88]	0.77	[0.74; 0.82]

*Note:* CI– confidence interval; GR– Growth rate; GRR– Growth rate ratio; IR– Incidence rate per 100 000 inhabitants; IRR– Incidence rate ratio; Model 1 analyses time between T1-T2, containing W1, W2 and W3; Model 2 analyses time between T2-T3, containing W3, W4 and W5; Model 3 analyses time between T1-T3, containing W1, W2, W3, W4 and W5

*T1 for Model 1 and Model 3 and T2 for Model 2

Table 2 shows the incidence trends during the study period. At the beginning of the tiered restrictions system implementation (T1), the incidence rate in the municipalities in moderate tier was 56.92/100 000 habitants (95%CI: 45.12; 71.81). Compared with the municipalities in moderate tier, those in high and very high tiers had a higher incidence rate, 2.66 times (95%CI: 1.96; 3.63) and 8.29 times (95%CI: 6.20; 10.90), respectively. Right after the tiered restrictions system implementation (Model 1, T1-T2), the incidence growth rate in municipalities in moderate tier were increasing 18% per week (95%CI: 7%; 30%). The municipalities in high tier were still increasing their incidence, but at a lower rate, by 6% (95%CI: -2%; 15%), while the very high tier immediately reversed the trend and started to decrease their incidence by 19% (95%CI: 14%; 24%). Compared with the moderate tier, both high and very high tiers exhibited a slowdown in the incidence growth rate, with the effect being more noticeable in the very high tier (GRR high tier: 0.90 [95%IC: 0.79; 1.02], GRR very high tier: 0.68 [95%IC: 0.61; 0.77]).

Two weeks after the tiered restrictions system implementation (Model 2, T2-T3), the incidence rate in the municipalities in moderate tier was 41.03/100 000 habitants (95%CI: 27.93; 60.27). Compared to this tier, the incidence rate was 3.44-fold (95%CI: 2.03; 5.85) in the municipalities in high tierand in municipalities in very high tier was 6.98-fold (95%CI: 4.31; 11.31). The incidence growth rate maintained the tendency observed in Model 1, with municipalities in moderate increasing 23% (95%CI: 12%; 34%), in high tier increasing at a lower rate, 8% (95%CI: 0%; 17%), and in very high tier decreasing by 3% (95%CI: -9%; 4%) per week. Compared with the municipalities in moderate tier, both high and very high tiers showed a deceleration in their growth rates (GRR high tier: 0.88 [95%CI: 0.78; 1.00]), GRR very high tier: 0.79 [95%CI: 0.71; 0.88]), although the very high tier had a more robust estimate.

Considering the entire study period, (Model 3, T1-T3), the incidence rate in the municipalities in moderate tier was 63.94/100 000 habitants (95%CI: 53.76; 76.05). Compared with moderate tier, the incidence rate in high and very high tier was 2.38 times (95%CI: 1.91; 2.98) and 5.82 times (95%CI: 4.71; 7.18), respectively. The incidence growth rate in municipalities in moderate tier was increasing by 15% (95%CI: 10%; 20%) per week and, comparing with this tier, we observed that municipalities in high tier were still increasing their incidence, but at a lower rate, by 6% (95%CI: 2%; 10%) per week. Whereas municipalities in very high tier decreased by 11% (95%CI: -13%; -8%). Compared with the moderate tier, the municipalities in both high and very high tiers slowed down their incidence growth rate, (GRR high tier: 0.92 [95%CI: 0.87; 0.98], GRR very high tier: 0.77 [95%CI: 0.74; 0.82]), with very high tier exhibiting a more pronounced decline. The sensitivity analyses associated with the study design showed consistent results in all models, namely the sensitivity analysis with the four tiers showed consistency with the trend observed in the main analysis, where the decrease in the growth rate increased as the tiers stringency increased (see Additional File 2). The models supporting our hypotheses had a better goodness of fit than the models corresponding to the null hypothesis. The AIC and BIC of the models can be seen in the Additional File 2.

## Discussion

The tiered restrictions system was implemented during the second COVID-19 wave in Portugal. We compared the COVID-19 growth rate across tiers to assess the effect of the tiered restrictions system. Our results indicated that the tiered restrictions system decelerated the growth rate faster in very high tier (i.e., stricter tiers) compared to the moderate tier. Although imprecise, a similar effect was found for the high tier, compared with the moderate tier. We also observed that right after the tiers implementation there was a lower albeit imprecise growth rate of high tier compared to the moderate tier. However, municipalities in very high tier already indicated a decrease. After two weeks, a difference was identified in very high tier, and after four weeks, that difference was larger and more precise. Municipalities in high tier also presented a decrease in both times, although with an imprecise 95%CI. These results are in line with Torres et al. which, in 2022, also showed that the tiered intervention decreased COVID-19 incidence in Portugal [21]. The effect of the tiered restrictions system was better observed in the overall model where the very high tier was the one with a more precise 95%CI in GRR compared to moderate tier, indicating a ladder effect, where more restrictions led to greater decrease. These findings support the thesis that time under stricter measures is important for COVID-19 infection reduction.

Very high tier seemed to have shown a faster deceleration in the growth rate. Similar results were found in studies analysing the tiered measures effect in other countries [7, 8, 16, 17]. Overall, these studies analysed changes in transmissibility and demonstrated that in regions under stricter tiers the effective reproduction number R(t) was reduced [7, 8] and there was also a reduction in infection rates [15], proportionally to tiers stringency level, as observed in our analysis. There was also evidence that restricting contact between people, especially to avoid confraternization between friends and relatives, played an important role in controlling COVID-19 infections [27]. In fact, similar to other tiered restriction systems implemented in other countries, the main differences regarding measures stringency between Portugal’s higher tiers (high vs. very high tiers) were also centred on controlling outdoor meetings, especially on weekends, and limiting the hospitality sector [7, 8, 15, 17]. Although there also seemed to be evidence that risk perception alone may have played an important role in people’s behaviour [28], as illustrated by the sensitivity analysis with the four tiers. In 2021 Manica et al. indicated that mobility in the stricter tier was reduced by over 50% compared to pre-pandemic values. The mobility indicators related to retail and recreation activities and public transportation were paralleled by an increase in the time spent at home [7], which could explain the growth rate reduction in stricter tiers. In 2022 Delusso et al. indicated that gradually increasing the stringency of the measures may support its compliance [29], which could have contributed to the effectiveness of the measures applied in those tiers, thus reducing infection rates.

While tiered measures might have been an interesting solution to flatten the unequal consequences the COVID-19 pandemic had on people, their success may also have been influenced by sociodemographic factors. A previous study identified sociodemographic and economic factors, such as occupation, household, income, and public transportation, which might explain COVID-19 dynamics in mainland Portugal [30].

Additionally, the unequal testing capacity across the country [1] could have also skewed the true interpretation of COVID-19 infection rates. This may have contributed to the increase in the incidence rate observed at the end of the study period, which also influenced the tiers assignment since this was based on municipalities incidence. The study period ended close to Christmas, when people were likely getting tested more to see family and friends or due to mandatory entry into certain places or events. Considering the results of our model and other studies regarding the tiered restrictions systems, these measures seemed likely to control the pandemic by slowing down transmission rates, contributing to social development, and normalisation of everyday life. There is evidence that stricter measures, namely lockdown scenarios, were more effective against COVID-19 severity by reducing hospitalisations and deaths [7, 8, 16]. Nonetheless, there is evidence that lockdowns not only lead to an exacerbation of inequalities due to socioeconomic losses but also increased mental stress in the population [31]. Thus, choosing these two approaches should be a trade-off between slowing and maintaining low transmission levels, considering the possible mitigation of social and economic effects of these public health measures [32]. The timing of the intervention was also an important factor to consider. The delay between the cases rising and the implementation of a strategy was shown to not only led to an exponential increase in the peak infections number but also delayed it proportionally to the time until the implementation [32–34]. The second wave of COVID-19 in Portugal started in September 2020, although the tiered restrictions system was only implemented nationwide in late November 2020. An earlier implementation of this system, closer to the beginning of the second wave, might have increased its effectiveness. The tiered restrictions system in Portugal was first enforced in certain municipalities that were above the ECDC incidence threshold for higher risk [9]. Studies analysing tiered measures have shown that the reduction observed in higher tiers may not be attributable only to the tier effect since the trend started to level before they were implemented [8]. However, in 2023 Davies et al. simulated the effect of tiered interventions and several kinds of lockdown scenarios throughout UK countries, having observed that the tiers-only scenarios decreased the COVID-19 transmissibility and kept it below the levels that would be expected with a lockdown. This suggested that the tiered restrictions system reduced the number of susceptible individuals in opposition to lockdowns as it lowered the infections in the short term, lowering the population’s immunity [16]. Thus, the decrease observed in very high tier might had a residual influence of this earlier intervention due to the accumulation of natural immunity [7]. Data from COVID-19 national serological surveys showed that between June 2020 and March 2021, the seroprevalence of COVID-19 infections in the Portuguese population increased from 2.9 to 15.5%, and in March 2021 13.3% was from post-infection [35, 36].

A curious result was an increase in the mean 7-day cumulative incidence observed in very high tier after W4 (14th December), which is almost 7 days after the two national holidays in this month (1st and 8th December). These two holidays preceding Christmas were close to the weekend and marked by heavy restrictions to avoid gatherings (see Additional File 1). After that, there was an increase in mobility, perhaps fuelled by the holiday season, which may have played a part in this infection’s increase [37]. Also, although more restrictions were in place during the holidays, some studies suggested that compliance with public measures may be reduced in stricter tiers [7, 29], which could impact the observed GRR. The vaccination campaign was identified as playing a role due to an adjustment of risk perception [29], as people could have lowered its protection due to the perceived protection of the vaccine. However, in Portugal, the COVID-19 vaccination only began in late December 2020, thus the results of this study were not influenced by the vaccination effect [38].

This study had several limitations that might influence its interpretation. Unequal geographic testing capacity could have led to measurement bias since the likelihood of getting a positive case increase [1]. Additionally, individuals living in higher tiers might be more likely to get tested due to the higher risk of infection. These limitations might have skewed our results by increasing the growth rate ratio observed in these tiers. Our study period also included the holiday season prior to Christmas, when people tend to move more, which may have led to higher testing increasing the infection rate towards the end of the study period. Still regarding the study period, the limited timeframe analysed could also have been a limitation since the results seemed to have shown that there is a lag in the effect of the measures, hence the long-term effect may have been greater than the one in our findings.

The study design analysed the average effect of a municipality being introduced in a tier and then being assessed every 14 days. It did not analyse the effect resulting from the change from a higher tier to a lower tier, which could have allowed us to analyse whether when a municipality moved to a lower tier, the less strict measures of that tier could have maintained the incidence at lower levels or whether the incidence would have increased again and force the municipality to return to a higher tier. Also, the tiers were assigned based on the 14-day cumulative incidence, which was also our measure of effect. Another limitation is that we did not assess the role of demographic and socioeconomic characteristics on the effect of the tiered measures. In particular, there was evidence that age, sex, and socioeconomic status were associated with compliance [39]. There was also evidence that compliance with public measures may be reduced in stricter tiers [7, 29], potentially influencing the GRR in the long-term. Additionally, there may have been differences between the economic sectors most prevalent in each municipality, i.e., there were regions in the country where the work nature did not allow remote working, which difficult the expected effect of measures to avoid work contacts between people. These limitations associated with factors related to measures compliance may have influenced COVID-19 transmission, hence biasing the association of the growth rate ratio with the tiers, underestimating our results. The adopted methodology did not allow us to pinpoint which specific restrictions contributed most to the slowdown of the growth rate, but this has not influenced our results since our aim was to access the growth rate ratio of moving from a lower to a higher tier. The methodology also did not comprehend the possible cross-border effects. For instance, a municipality in moderate tier that shared borders with a municipality in one of the very high tier may have indirectly benefited from the restrictive measures imposed in the latter, i.e., reduction of inter-municipality mobility, resulting essentially from curfews and teleworking.

Despite the limitations, this study had several strengths which bring information to the decision-making. It adds to the evidence of the effect of tiered systems implemented in several countries by analysing its effect in Portugal. Our models considered different periods, enabling us to observe the effects on COVID-19 incidence growth rate between different time periods. Of the European countries we could find evidence from the tiered system intervention, Portugal was the only one that used the ECDC risk criteria alone for tiers assignment and analysed COVID-19 incidence growth rate variation in each tier. This study adds to the existing literature on tiered measures, providing evidence related to these criteria.

## Conclusion

Our findings support that the tiered restrictions system decelerated the growth rate in the very high tier, thus controlling the transmissibility of COVID-19 infections. Municipalities in high tier also showed a deceleration compared with moderate tiers, although with imprecise values. Hence, our results seemed to show that increasing measures’ stringency contributed to reduce the growth rate of COVID-19 cases, which places these tiered systems as an alternative to nationwide lockdowns, and part of an effective strategy for reducing geographical differences in transmission. Also, although vaccination is already a reliable tool to fight COVID-19, evidence regarding non-pharmaceutical interventions remains relevant as it might be needed in the future, as new variants emerge or even for another respiratory virus outbreak. Our results also showed a lag between tiered restriction system implementation and when their effects began to be visible, which highlights the importance of timely and assertive measures implementation. However, studies analysing a broader period are needed to understand the effectiveness of the tiered restrictions system as a reliable alternative for pandemic control.

### Electronic supplementary material

Below is the link to the electronic supplementary material.

Supplementary Material 1

Supplementary Material 2

## Data Availability

The dataset analysed during the current study are available in the GitHub repository, https://github.com/dssg-pt/covid19pt-data/. Data on population estimates for each municipality is available in Statistics Portugal, https://www.ine.pt; and data on tiers assigned to each municipality is available in COVID-19 reports, https://covid19.min-saude.pt/relatorio-de-situacao/.

## Abbreviations

NPIs: Non-pharmaceutical interventions
ECDC: European Centre for Disease Control
DGS: Portugal Directorate-General of Health
GR: Growth rate
GRR: Growth rate ratio
CI: Confidence interval
