# Supplementary material for: Is a tiered restrictions system an effective intervention for COVID-19 control? Results from Portugal, November-December 2020
Source: BMC Public Health. 2024 Apr 4;24:956. doi: 10.1186/s12889-024-18369-1 (PMC10993531; doi:10.1186/s12889-024-18369-1)
Supplement: Supplementary file 1 — Supplementary Material 1 [file 12889_2024_18369_MOESM1_ESM.pdf]

Additional File 1 – Measures enforced in each tier

Measures enforced nationwide

- Mandatory confinement for patients with COVID-19 and people under active surveillance.
- Mandatory use of masks or face shields to access or stay in workplaces whenever recommended physical distance proves impractical.
- Body temperature measurement using non-invasive instruments could have been required at workplaces, educational institutions, prisons, residential structures, public transports, services, institutions, commercial, cultural and sports.
- Diagnostic tests for COVID-19 could have been required when accessing health facilities, residential structures, residential structures for the elderly, units of integrated continuous care of the National Network of Integrated Continuous Care and other responses dedicated to the elderly, as well as children, young people and people with disabilities, educational establishments, professional establishments, and when entering and leaving the national territory – by air or sea – and other places, as determined by the National-Directorate of Health. Mandatory negative RT-PCR COVID-19 test results in less than 72h on departure and arrival at the airport.
- Day off was granted for all public administration on 30th November and 7th December, and academic activities were suspended on the same days due to the two public holidays on the 1st and 8th of December.
- Prohibition of movement outside the municipality of residence between 11 pm on 27th November 2020 and 5 am on 2nd December 2020, and between 11 pm on 4th December 2020 and 11:59 pm on 8th December 2020, except for health reasons or other reasons of imperative urgency. Exceptions to this measure can be found in the official referred documents.(7,12)
- Closure of establishments for recreational purposes, leisure, and fun activities except for those integrated in tourist and local accommodation establishments, for the exclusive provision of services to the respective guests.
- Maximum indicative capacity of 0.05 people per m2 at all locations open to the public except for service provision establishments.
- Closure of catering establishments or similar at 1 am, without new admissions after 12 am. Capacity restriction to 50% of the capacity of each establishment, with groups limited to 6 people unless they belonged to the same household. Whenever located up to 300 meters from a school, establishments would have to close at 8 pm, and groups were limited to 4 people unless they belonged to the same household. In food and beverage consumption areas (food-courts) of commercial complexes, groups of more than 4 people were not allowed unless they belonged to the same household.
- Closure of bars and other drinking establishments without a show and drinking establishments with a dance floor.
- Prohibition of alcoholic beverages sales in service areas and gas stations, and from 8 pm onwards, in retail establishments, including supermarkets and hypermarkets, and home deliveries, directly or through an intermediary or take-away services. Consumption of alcoholic

beverages was prohibited in outdoor spaces with public access and public roads, except for the outdoor spaces of food and drink establishments duly licensed for this purpose.

- Private vehicles with a capacity of more than 5 seats could only circulate with 2/3 of their occupancy unless they all belonged to the same household.
- Competent local authority of each cemetery should adopt measures to guarantee the absence of crowds of people and to control safety distances (e.g., setting a maximum attendance limit). Nevertheless, those measures could not result in the impossibility of the presence at the funeral of a spouse or partner, ascendants, descendants, or relatives.
- Fairs and markets were allowed to operate in accordance with specific rules.
- Hairdressing salons, barbers, beauty institutes, tattoo and body piercing establishments or studios were allowed to function only by appointment.

Moderate tier

In addition to the measures enforced nationwide, the municipalities in this tier were also enforcing the following measures:

- Closure of catering or similar and cultural and sports establishments between 8 pm and 11 pm. Municipality mayors could decide to close these establishments within this interval, with the favourable opinion of the local health authority and security forces.
- Prohibition of celebrations and other events involving a gathering of more than 6 people unless they belonged to the same household. Religious ceremonies, weddings, Christian ceremonies (no more than 50 people) and corporate or cultural events were subject to specific Directorate-General of Health (DGS) guidelines.

High tier

In addition to all the previous measures, municipalities in high tier were also enforcing the following measures:

- Mandatory curfew, between 11 pm and 5 am, seven days a week (exceptions as described for the nationwide measures also apply). Roads and rail traffic were closed for public health, safety or traffic flow reasons, and certain types of vehicles had movement restrictions during this period.
- Closure of all retail and service establishments, as well as those located in commercial complexes, until 11 pm, except for:
  - Catering establishments had to close until 10:30 pm. After that, they could stay open until 1 pm exclusively for home delivery.
  - When intended for federated sports, cultural and sports facilities had to close until 10:30 pm.
- Prohibition of fairs and markets, except when authorised by the mayor.
- Prohibition of celebrations and other events involving a gathering of more than 6 people, unless they belonged to the same household, except for religious ceremonies, cultural shows, or scientific events.

- Mandatory teleworking, regardless of the employment relationship and whenever the functions allowed it.

Very or extremely high tiers

In addition to all the previous measures, municipalities in very or extremely high tier were also enforced to adopt the following measures:

- Mandatory curfew, between 1 pm and 5 am, on weekends and holidays. (The exceptions were the same considered nationwide, including trips to grocery stores, supermarkets and other establishments selling food and hygiene products for people and animals, as well as trips to access events and cultural facilities).
- General duty of home confinement, every day, outside the period between 11 pm and 5 am, as well as on Saturdays, Sundays, and public holidays between 5 am and 1 pm. (The exceptions were the same as described in the nationwide measures)
- Suspension activities in retail trade and service provision establishments on Saturdays, Sundays, and holidays, outside the period between 8 am and 1 pm, and on 30th November and 7th December, outside the period between 8 am and 3 pm. In addition to the exceptions already mentioned, establishments selling food with a door to the street up to 200 m2 in area and gas stations were also closed.
- Closure of commercial establishments from 3 pm until 5 am on the eve of the two public holidays, mentioned in the nationwide measures.
