# Supplementary material for: Is a tiered restrictions system an effective intervention for COVID-19 control? Results from Portugal, November-December 2020
Source: BMC Public Health. 2024 Apr 4;24:956. doi: 10.1186/s12889-024-18369-1 (PMC10993531; doi:10.1186/s12889-024-18369-1)
Supplement: Supplementary file 2 — Supplementary Material 2 [file 12889_2024_18369_MOESM2_ESM.pdf]

Additional File 2 – Results of the sensitivity analyses

Four tiers analysis						
**Parameters**	Model 1 *(T1-T2)*		Model 2 *(T2-T3)*		Model 3 *(T1-T3)*	
	Estimate	*95% CI*	*Estimate*	*95% CI*	*Estimate*	*95% CI*
*IR in Moderate tier in the first moment**	55.99	[44.63; 70.24]	41.15	[28.02; 60.46]	62.87	[53.05; 74.51]
IRR High tier vs moderate tier	2.74	[2.03; 3.72]	3.45	[2.03; 5.87]	2.42	[1.94; 3.01]
IRR Very high tier vs moderate tier	6.26	[4.62; 8.49]	5.74	[3.38;9.75]	4.54	[3.64; 5.67]
IRR Extremely high tier vs moderate tier	13.15	[9.34; 18.51]	10.23	[5.30; 19.74]	9.43	[7.26: 12.24]
GR Moderate tier	1.17	[1.07; 1.29]	1.22	[1.12; 1.34]	1.15	[1.10;1.20]
GR in High tier	1.05	[0.97; 1.14]	1.08	[0.99;1.17]	1.05	[1.02; 1.10]
GR in Very high tier	0.88	[0.81; 0.96]	1.01	[0.93; 1.10]	0.96	[0.92;1.00]
GR in Extremely high tier	0.76	[0.68; 0.85]	0.90	[0.79; 1.02]	0.82	[0.78; 0.87]
GRR High tier vs moderate tier	0.89	[0.79; 1.02]	0.88	[0.78; 1.00]	0.92	[0.87; 0.98]
GRR Very high tier vs moderate tier	0.75	[0.66; 0.86]	0.82	[0.73; 0.93]	0.84	[0.79; 0.89]
GRR Extremely high tier vs moderate tier	0.65	[0.56; 0.75]	0.74	[0.63; 0.86]	0.72	[0.67; 0.77]

Note: CI – confidence interval; GR – Growth rate; GRR – Growth rate ratio; IR – Incidence rate per 100 000 inhabitants; IRR – Incidence rate ratio; Model 1 analyses time between T1-T2, containing W1, W2 and W3; Model 2 analyses time between T2-T3, containing W3, W4 and W5; Model 3 analyses time between T1-T3, containing W1, W2, W3, W4 and W5; *T1 for Model 1 and Model 3 and T2 for Model 2.

14-day cumulative incidence analysis						
**Parameters**	Model 1 *(T1-T2)*		Model 2 *(T2-T3)*		Model 3 *(T1-T3)*	
	Estimate	*95% CI*	*Estimate*	*95% CI*	*Estimate*	*95% CI*
*IR in Moderate tier in the first moment**	117.76	[97.43; 142.34]	92.66	[70.42; 121.92]	130.88	[113.36; 151.10]
IRR High tier vs moderate tier	2.34	[1.83; 2.99]	3.17	[2.20; 4.58]	2.18	[1.83; 2.59]
IRR Very high tier vs moderate tier	6.37	[5.05; 8.03]	8.10	[5.79; 11.32]	5.57	[4.72; 6.59]
GR Moderate tier	1.16	[1.08; 1.24]	1.17	[1.10; 1.25]	1.11	[1.08; 1.15]
GR in High tier	0.94	[0.85; 1.03]	0.90	[0.82; 0.97]	0.95	[0.91; 0.99]
GR in Very high tier	0.77	[0.71; 0.84]	0.75	[0.72; 0.84]	0.82	[0.78; 0.85]
GRR High tier vs moderate tier	1.09	[1.03; 1.15]	1.05	[1.00; 1.11]	1.06	[1.03; 1.09]
GRR Very high tier vs moderate tier	0.89	[0.85; 0.94]	0.91	[0.87; 0.95]	0.91	[0.89; 0.93]

Note: CI – confidence interval; GR – Growth rate; GRR – Growth rate ratio; IR – Incidence rate per 100 000 inhabitants; IRR – Incidence rate ratio; Model 1 analyses time between T1-T2, containing W1, W2 and W3; Model 2 analyses time between T2-T3, containing W3, W4 and W5; Model 3 analyses time between T1-T3, containing W1, W2, W3, W4 and W5; *T1 for Model 1 and Model 3 and T2 for Model 2.

Results of the sensitivity analysis for goodness-of-fit assessment – model selection:

Analysis	Model 1	Model 2	Model 3
Principal analysis	AIC = 7914.5<br>BIC = 7953.1	AIC = 7950.2<br>BIC = 7988.9	AIC = 13221.9<br>BIC = 13264.6
Analysis without the interaction between tier and week	AIC: 7958.2<br>BIC: 7987.2	AIC: 7962.8<br>BIC: 7991.8	AIC: 13309.9<br>BIC: 13341.9
Analysis considering municipality as a fixed variable	AIC: 8060.122<br>BIC: 8093.923	AIC: 8177.807<br>BIC: 8211.608	AIC: 13560.57<br>BIC: 13597.95

Note: AIC: Akaike Information Criterion; BIC: Bayesian information criterion.
